# What is the effect of presenting evidence of the mental vs physical health benefits of quitting smoking on motivation to stop smoking? An online randomised controlled experiment

**DOI:** 10.1186/s12889-025-22795-0

**Published:** 2025-07-03

**Authors:** Katherine Sawyer, Adham Hanafi, Tom P. Freeman, Chloe Burke, Sally Adams, Paul Aveyard, Pamela Jacobsen, Gemma Taylor

**Affiliations:** 1 Addiction and Mental Health Group (AIM), Department of Psychology, University of Bath, 10 West, Bath, BA2 7AY UK; 2 Bath Centre for Mindfulness and Compassion, Department of Psychology, University of Bath, 10 West, Bath, BA2 7AY UK; 3 Institute for Mental Health, School of Psychology, University of Birmingham, Birmingham, UK; 4 NIHR Oxford Health Biomedical Research Centre, Warneford Hospital, Warneford Lane, Oxford, OX3 7JX UK; 5 Nuffield Department of Primary Care Health Sciences, University of Oxford, Radcliffe Observatory Quarter, Woodstock Road, Oxford, OX2 6GG UK; 6 NIHR Oxford Biomedical Research Centre, John Radcliffe Hospital, Oxford, OX3 9DU UK; 7 NIHR Oxford and Thames Valley Applied Research Collaboration, Radcliffe Observatory Quarter, Woodstock Road, Oxford, OX2 6GG UK; 8 Centre for Public Health, Population Health Sciences, Bristol Medical School, University of Bristol, Canynge Hall, 39 Whatley Road, Bristol, BS8 2PS UK

**Keywords:** Smoking, Cessation, Public health, Mental health, Tobacco warning labels

## Abstract

**Objectives:**

Tobacco warning labels usually present information about the consequences of smoking but gain-framed messages could be a novel strategy to prevent wear out effects. Communicating the mental health benefits of stopping smoking could motivate cessation. We assessed whether such messaging was more effective at motivating cessation than blank labels and labels communicating the physical health benefits of cessation, and if there were any differences between those with mental ill health, or not.

**Design:**

An online randomised parallel experiment. Participants were randomly allocated using online randomised function to a condition, stratified by mental health status.

**Setting:**

Online survey platform, Qualtrics.

**Participants:**

People who smoke tobacco weekly, above the age of 18.

**Intervention:**

Gain-framed tobacco packaging labels with three conditions: i) mental health labels; ii) physical health labels; iii) blank labels. Each condition consisted of four labels, viewed for at least 10 seconds each.

**Primary outcome measure:**

Motivation to stop smoking, measured using the Motivation to Stop Scale (MTSS) at baseline and follow up immediately after viewing the labels. 631 people who smoke tobacco weekly were randomised. Compared to the blank labels, those who viewed mental health labels had higher post-viewing MTSS scores (β = 0.18, *p* = 0.003, 95% CI [0.07, 0.30]). There was no evidence for a difference in post-viewing MTSS scores between the mental health label and physical health label (β = 0.07, *p* = 0.16, 95% CI [-0.03, 0.18]) conditions. These findings did not differ based on mental health status or anhedonia symptoms.

**Conclusions:**

Labels on tobacco packaging that promote the mental health benefits of stopping smoking could motivate cessation and may be as effective as gain-framed physical health labels.

**Trial registration:**

clinicaltrials.gov NCT06762756, on 19/12/2024. Retrospectively registered.

**Supplementary Information:**

The online version contains supplementary material available at 10.1186/s12889-025-22795-0.

## Introduction

Tobacco smoking is a leading cause of preventable death, disease and health inequality [1]. Although smoking prevalence in high-income countries is reducing in the general population, smoking remains disproportionately high in people with mental health problems. People with mental health problems are twice as likely to smoke than the general population, leading to a reduced life expectancy in people with severe mental illness [2, 3].

Most countries require tobacco packets to feature health warnings. Graphic warnings about smoking's physical effects can encourage quit attempts and heighten harm perception [4–6]. Loss-framed messages highlighting health risks effectively deter smoking initiation [7]. However, their impact may diminish over time, necessitating periodic updates to labels for sustained effectiveness [4].

Gain-framed messages could be a novel strategy for smoking cessation but there is limited evidence on their effectiveness [8]. Initial research suggests that cigarette packs with gain-framed messages can reduce cigarette pack appeal, but there are no differences between gain-framed and loss-framed smoking messages [9]. Gain-framed messages are more effective than loss framed messages at promoting abstinence in people who smoke who are highly dependent, but not in people who smoke who are less-dependent [10]. In Canada, packaging inserts highlighting benefits like financial savings and improved health increased quit attempts [11]. However, research largely focuses on physical health benefits, with limited studies on using gain-framed messages to communicate mental health benefits of quitting.

Observational studies suggest quitting smoking improves mental health, a change not seen in those who continue smoking, with evidence of a causal link [12, 13]. Concerns exist about stigma from labels highlighting smoking’s negative mental health effects [14]. Communicating that quitting improves mental health is acceptable to people who both smoke and have mental health issues and could be a strategy to increase motivation to quit [15]. Steinberg et al. [16] found no difference between gain-framed mental vs. physical health messages in perceived effectiveness but noted higher motivation to quit in those with mental health histories when exposed to mental health-focused posters. However, the impact of such messages on large-scale interventions like warning labels remains unknown. This study examines whether presenting mental health benefits of quitting on tobacco packaging motivates quitting.

This study explores whether gain-framed messages about mental health benefits are as effective in motivating smoking cessation as those highlighting physical health benefits. By comparing gain-framed mental and physical health messages, rather than loss-framed warnings, we aim to isolate the impact of the message topic (mental vs. physical health) rather than the framing.

This study examines whether the impact of mental health labels varies by mental health status. Evidence suggests people who both smoke and have mental health problems may respond differently to tobacco warnings [17, 18]. Media campaigns showing mental health benefits of quitting increased quit intentions and attempts in people who both smoke and have mental health issues compared to those who smoked but didn’t have mental health issues [19]. Steinberg et al., [16] found mixed results, with differences linked to lifetime anxiety/depression history but not current symptom severity .

As a secondary question, we assessed if mental health symptoms moderate responses to mental health labels. Anhedonia, the inability to experience joy or pleasure and a key symptom of psychiatric disorders such as depression, is strongly linked to higher smoking rates and difficulty quitting [20–22]. Considering limited research on how specific symptoms affect cessation message responses [16, 19], we explored whether anhedonia moderates the effect of mental health messaging on motivation to quit.

An extensive body of literature investigates the impact of loss-framed physical health tobacco warning labels, with many different outcomes assessed across the literature. Noar et al. [5] synthesised these outcomes into five constructs within the “Message Impact Framework” [5]. The framework includes attention and recall, warning reactions, attitudes and beliefs, intentions and behaviour [5]. This study uses the Message Impact Framework to inform secondary outcomes to examine the impact of presenting mental health benefits on tobacco packaging.

### Primary research question

What is the effect of presenting evidence that quitting smoking is associated with mental health benefits on motivation to stop smoking, compared to active and non-active control conditions?

### Secondary research questions

1. Does mental health status moderate the effect of positive mental health labels on motivation to stop smoking?
2. Does anhedonia moderate the effect of positive mental health labels on motivation to stop smoking?
3. What is the effect of presenting evidence that quitting smoking is associated with mental health benefits on each component in the Message Impact Framework, compared to active and non-active control conditions?

## Methods

This study was retrospectively registered on clinicaltrials.gov (NCT06762756) on 19/12/2024. A protocol for this study was pre-registered on the Open Science Framework (OSF), prior to data-collection (10.17605/OSF.IO/3C75J). Ethical approval was granted by University of Bath Research Ethics Committee on 18th November 2021 (PID: 21–244). This study adheres to CONSORT guidelines, see CONSORT checklist.

### Design

This study was an online randomised experiment using the survey platform Qualtrics and used a cross-sectional experimental between-subjects design, with 3 groups: i) mental health labels; ii) physical health labels; iii) blank labels. Each group was stratified by mental health status, so there were balanced numbers of people with and without mental health problems in each group. There were four labels per condition. There were no changes to methods or outcomes after study commencement.

Gain-framed physical health labels not in circulation were selected as one of the control conditions to provide a comparable positive message to contrast with the positive mental health labels. Since no gain-framed physical health labels are currently approved for use on tobacco packaging in the UK, and tobacco packaging does not provide information about the benefits of quitting smoking, this choice provided a necessary point of comparison. Additionally, to ensure the highest level of experimental rigor, a blank control condition was included as a true null control to isolate the effect of the new stimuli, gain-framed mental health labels, on motivation to quit smoking. This approach has been used in previous research on health behaviour, including studies on e-cigarettes [23].

Current physical health warnings are not an optimal control condition for this initial proof of concept study for two key reasons. Firstly, their content differs in two ways from our main experimental condition of interest (i.e., loss-based physical health messages versus gain-based mental health messages). This would have compromised our ability to isolate the effects of interest in this study, as we could not have known if differences between conditions were driven by the content (mental or physical health) and/or the framing (negative versus gain based). Secondly, these physical health messages are already in circulation, creating familiarity to participants. This limits the extent to which they can be considered a suitable control condition where experimental and control conditions are closely matched (e.g., due to familiarity). Overall, using both blank labels and positive physical health labels allows us to isolate any true effects of the experimental manipulation rigorously, enabling us to rule out other factors such as negative framing, graphic nature, or familiarity with labels already in circulation.

### Participants

Participants recruited into the study were people who self-reported smoking tobacco weekly, aged at least 18 years, who could read English. Smoking status was determined by a self-report of smoking cigarettes (manufactured or hand-rolled) at least weekly. The focus of this study was on cigarette smoking, use of other tobacco products was not specified as an inclusion criterion or in the outcome assessment. The target sample size was 636, based on power analysis for the primary research question using G*Power, with 90% power to determine an effect size (f2) of 0.02 on our primary outcome at an alpha level of 0.05. The effect size used in this power calculation was not based on prior research due to the limited available literature using positive quit messages on tobacco packaging. A small effect size was chosen based on standard cutoffs because of the relatively novel research question and consideration that effect sizes from such public health interventions are typically quite small.

### Procedure

Participants were recruited between September 2022 and February 2023 using online research recruitment platforms (e.g. Prolific and MQ Participate) and the student research participation scheme at the University of Bath. We did not actively detect or include questions for bot detection in our survey. Prolific has an inbuilt participant verification system, but other online platforms do not. Participants were provided with a Qualtrics study link which presented the participant information sheet and eligibility criteria. After assessing eligibility and providing informed consent via the online portal, demographic information and baseline measures were collected. Participants were then randomised to one of the three experimental conditions. In each condition, participants viewed four labels of both images and text, one at a time for a minimum of 10 seconds each. After viewing all four labels, a ‘next’ button appeared, and participants could proceed to answer all outcome measures (see Supplementary Table 1). Participants were then directed to a debrief page and given the option to be redirected to a separate survey to enter a £50 prize draw by providing a name and email address. There were no anticipated harms or unintended effects in the study.

### Randomisation procedure

Participants were randomised using the embedded function within Qualtrics according to a 1:1:1 ratio into the three experimental conditions. Randomisation was stratified by mental health status, using the Qualtrics quota function, based on scoring above or below on clinical cut-off scores on measures of anxiety and depression ((≥ 8) on the Generalised Anxiety Disorder scale (GAD-7) [24] or (≥ 10) on the Patient Health Questionnaire (PHQ-9) [25, 26].

### Allocation concealment

Randomisation to experimental condition was automated by the online system and was not accessible to any member of the research team, ensuring good allocation concealment.

## Materials and measures

Four labels for each experimental condition were designed by the study team. Labels were informed by previous literature demonstrating the benefits of quitting smoking [12] and NHS campaigns on the physical health benefits of quitting smoking. Images for both conditions were based on the style of the images used in the NHS physical health quit smoking campaigns and generic stock images of wellbeing. These initial labels and study materials were presented to a patient and public involvement (PPI) group in a series of online meetings. The PPI group consisted of people, all of whom had experience of mental health problems and smoking. The group were asked for feedback on the images, phrasing of messages for each condition, and outcome measures. For a full summary of PPI feedback see OSF protocol supplementary materials 1 (10.17605/OSF.IO/3C75J).

**Fig. 1:**
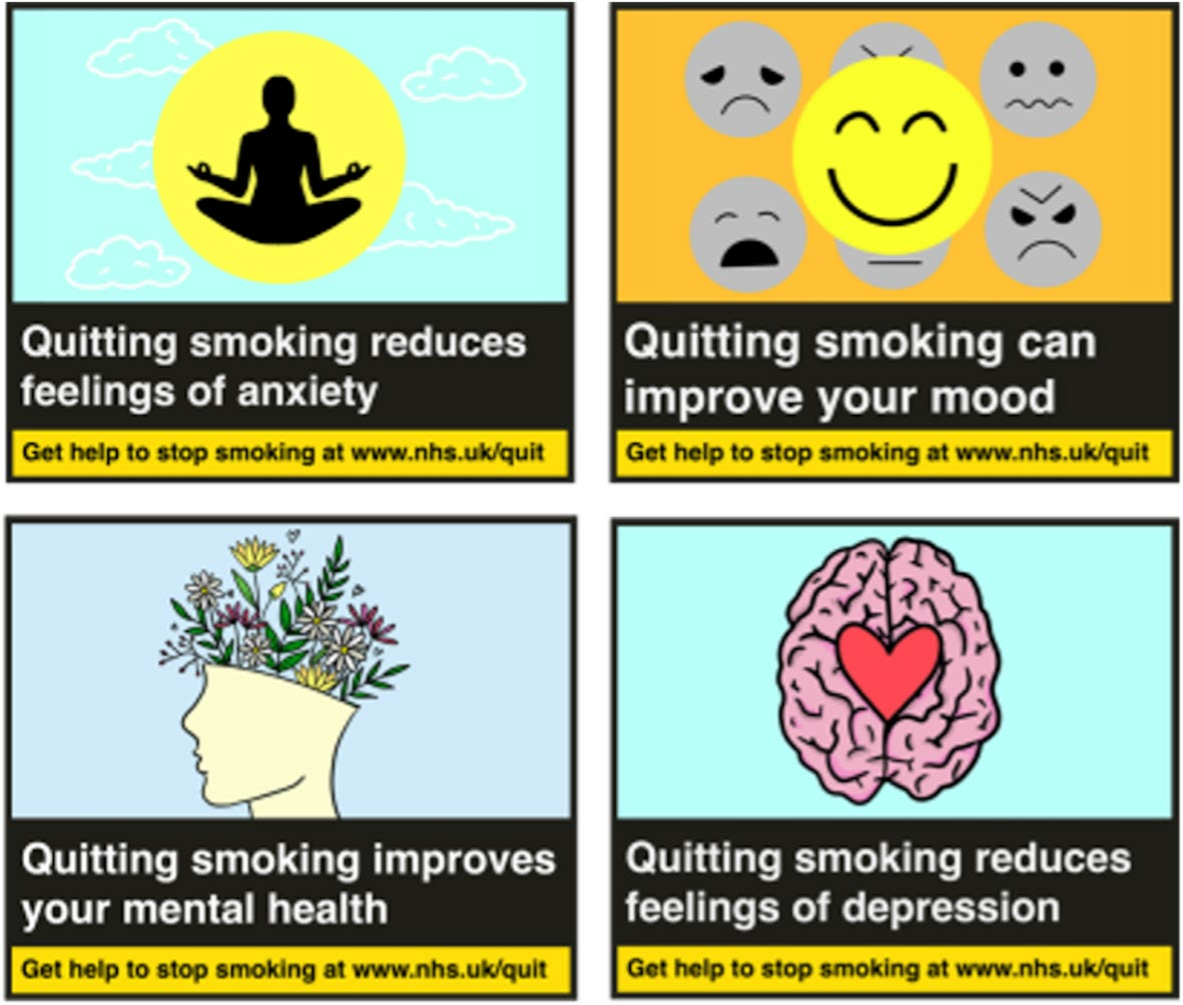
Experimental stimuli: Mental health labels (experimental condition)

**Fig. 2:**
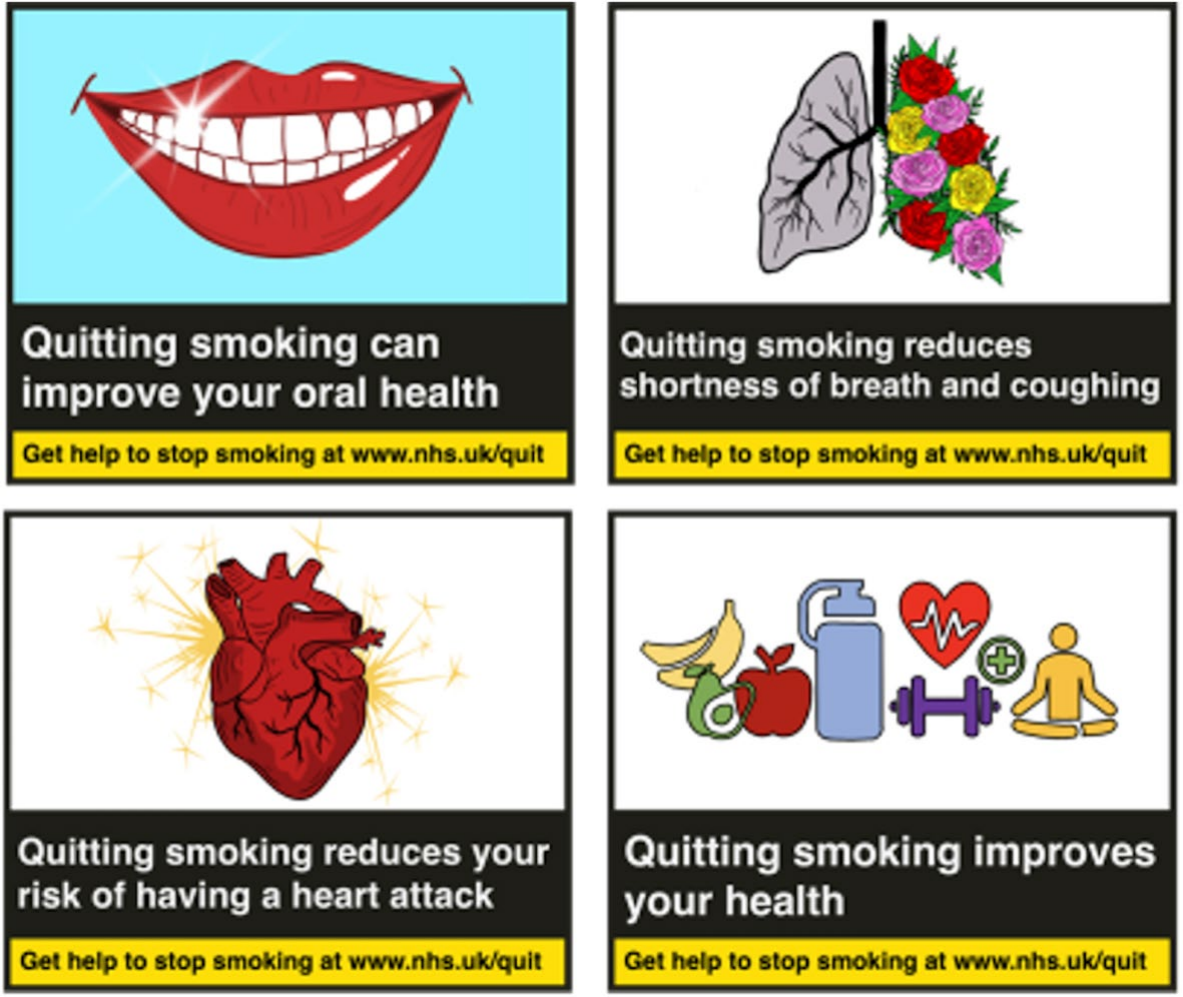
Experimental stimuli: Physical health labels (active control)

**Fig. 3:**
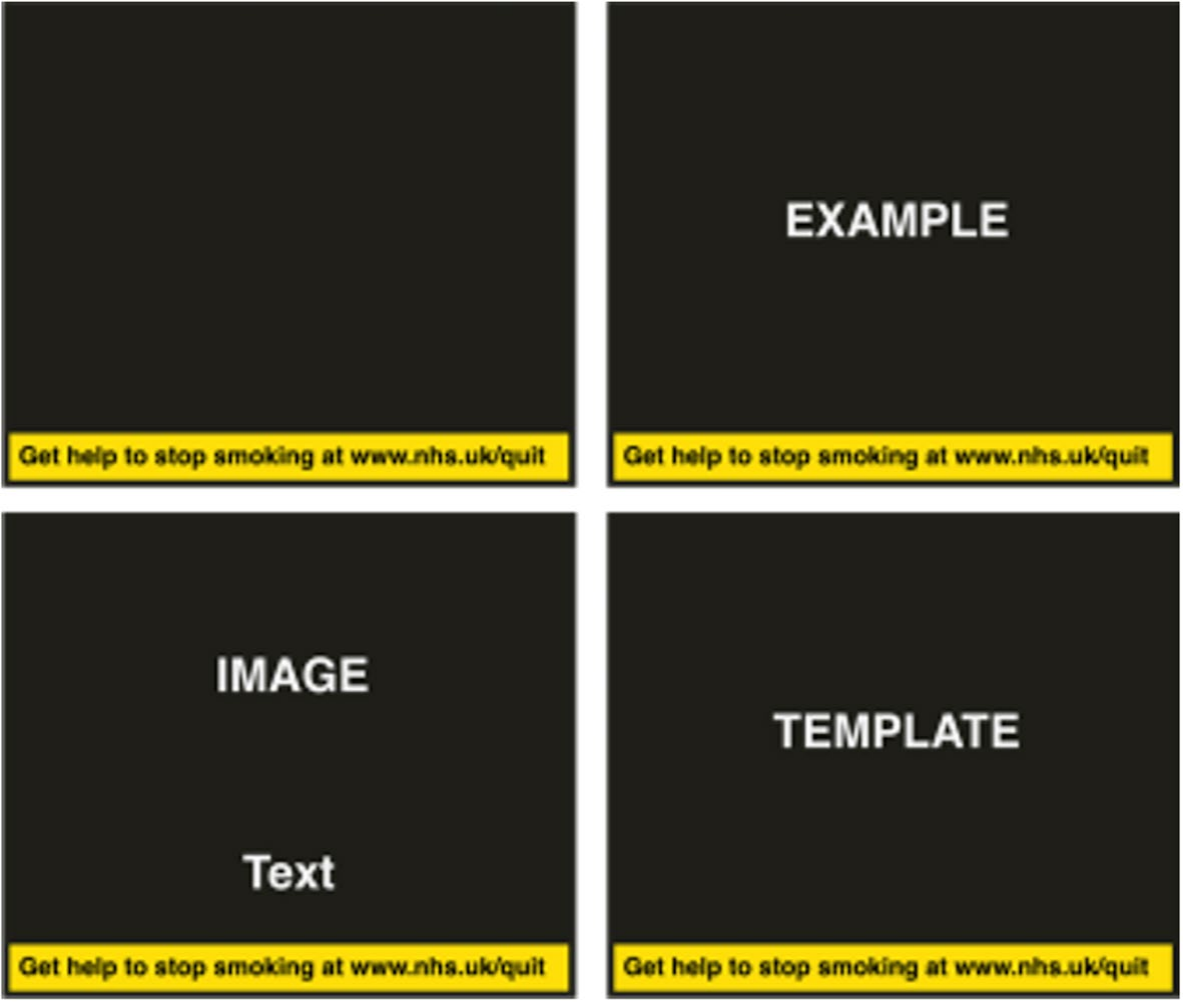
Experimental stimuli: Blank labels (non-active control)

Labels were formatted in a stacked format, as published on tobacco products and in accordance with the Great Britain Tobacco Packaging Guidance [27]. The final experimental stimuli are presented in Figs. 1, 2 and 3, Labels were presented consecutively (top left, top right, bottom left, bottom right). As a result of PPI feedback, labels were presented with the following caption “You may see images containing health-related messages, or blank templates, either is fine. This is normal and your participation, regardless of what type of images you see, is vital to the success of this project.”

### Demographic measures, mental health, and smoking information

Smoking Status: Smoking status was measured using the question “How often do you smoke cigarettes (manufactured or hand-rolled)?” On a scale of: ‘Every day’, ‘Every week’, ‘Less than every week’, ‘Not at all’. Participants who selected ‘Every week’ or ‘Every day’ were categorised as people who smoke and eligible for the study.

Nicotine Dependence: Nicotine dependence was assessed using The Fagerström Test of Nicotine Dependence (FTND), which has a range of 0–10, where higher scores indicated higher dependence [28]. Participants were also asked how many quit attempts they had made in the past six months.

Mental Health Status: We determined whether people had anxiety or depression from their scores on GAD- 7 or PHQ- 9 using their respective case thresholds (≥ 8 on the GAD-7 [24] or ≥ 10 on the PHQ-9 [25, 26]). The GAD-7 [24] and PHQ-9 [25] are not diagnostic tools but instead measure severity of symptoms, with higher scores indicating higher severity.

Anhedonia: Anhedonia was measured using the Snaith-Hamilton Pleasure Scale (SHAPS) [29]. The SHAPS is a well-validated, 14 item, measure of anhedonia [21, 29, 30]. The SHAPS has a range of 0–14 with scores ≤ 3 seen as a clinical level of anhedonic traits, and higher scores indicating lower levels of anhedonic traits [29].

### Primary outcome measure

Motivation to Stop: We used the Motivation to Stop Scale, (MTSS; [31]) which has been psychometrically tested and strongly predicts quitting behaviour. The MTSS asks participants “Which of the following best describes you?” with the following ordinal scale: 1) “I don't want to stop smoking”; 2) “I think I should stop smoking but don't really want to”; 3) “I want to stop smoking but haven't thought about when”; 4) “I REALLY want to stop smoking but I don't know when I will”; 5) “I want to stop smoking and hope to soon”; 6) “I REALLY want to stop smoking and intend to in the next 3 months”; 7) “I REALLY want to stop smoking and intend to in the next month”.

### Secondary outcomes measures

The secondary outcomes were based on The Message Impact Framework for research on cigarette pack warnings, a consensus of different measures to guide warning label research [5]. Secondary outcome measures included intention to quit smoking, quitting self-efficacy, smoking beliefs, attention, affective reactions (valence, arousal and dominance), and believability.

Intention to Quit: Participants were asked their intention to quit smoking “Are you planning to quit smoking within the next month?” using a 10‐point visual analogue scale, where 1 indicates low intention to quit and 10 indicates higher intention to quit smoking. This scale was adapted from work by Adams et al. [32].

Quitting Self-Efficacy: To measure participants’ self-efficacy to quit smoking, participants responded to the questions “Overall, how confident are you that you can stop smoking within the next month?” on a 5-point scale 1 (‘not at all’) to 5 (‘completely confident’) and “For me cutting down on the number of cigarettes that I smoke in the next month would be….” On a scale of 1 (‘very difficult’) to 5 (‘very easy’). The mean score across the two items was calculated, with a higher score indicating higher self-efficacy. This scale was adapted from one used by Harris et al. [33] and Sillero-Rejon et al. [34].

Smoking Beliefs: Participants rated their agreement to the statements “smoking helps people relax”, “smoking helps to reduce stress”, “smoking helps to keep weight down” “smoking increases social comfort” “those who smoke are more popular” and “second hand smoke is not harmful” using a 5-point scale from 1 (‘not at all’) to 5 (‘a lot’). The mean score across the five items was calculated, with a higher score indicating more favourable beliefs towards smoking [5, 35].

Attention: The extent to which the labels attracted or grabbed the participants attention was measured using measures from Cantrell et al., [37]. Participants rated two statements "these labels are worth remembering" and "these labels grabbed my attention" on a 5-point scale from 1 (‘not at all’) to 5 (‘a lot’), mean score across the two items was calculated, with a higher score indicating greater attention.

Affective Reactions: To measure affective reactions to the health labels we used the Self-Assessment Manakin (SAM, [36]). Participants rated their affective reactions: valence, arousal, and dominance on a 9-point visual analogue scale. Scales for valence range from 1 'unpleasant' to 9 'pleasant', arousal ranged from 1 'calm' to 9 ‘excited’, and dominance ranged from 1 ‘controlled’ to 9 ‘in control’ with 5 as neutral for all items. This measure is used instead of measures of fear and disgust in the Label Impact Framework as it is more relevant to the positive labels and has been used in other studies assessing emotional responses to tobacco warning labels [17].

Believability: Perceptions of believability of the health label were measured using the question “How believable are these health labels?”, participants rated this on a 5-point scale from 1 (‘not at all’) to 5 (‘a lot’) [5, 37].

### Statistical analysis

All analysis was conducted in R Studio (version 2023.12.1 + 402). Data and scripts available on OSF (10.17605/OSF.IO/3C75J) and University of Bath Data Archive. We assessed the impact of label condition on motivation to stop smoking with a regression equation predicting post MTSS from baseline MTSS and label condition.

We examined whether having a common mental illness moderated the effect of label condition using the same regression model as above but adding terms for mental illness and the interaction between that and label condition. As people with mental illness are more dependent on smoking than people who smoke who don’t have mental illness, we ran a sensitivity analysis additionally adjusting this model for baseline FTND score (Supplementary Table 3). For secondary research question two, the model included baseline motivation to stop smoking, label condition, anhedonia score, and an interaction term for anhedonia and label condition. Anhedonia was mean centred to overcome violations of multicollinearity in this model. For all models, visual inspection of Q-Q plots prompted concerns of violation of normality of residuals, so each model was bootstrapped 2000 times.

We examined the effect on the Message Impact Framework variables using separate multiple linear regressions for intention to quit smoking, quitting self-efficacy and smoking beliefs. The models included label condition and baseline score for each outcome; these models had non-normally distributed residuals so were bootstrapped with 2000 repetitions. For attention, affective reactions (valence, arousal, and dominance), and believability the models included label condition only. These models had non-normally distributed residuals so were bootstrapped with 2000 repetitions. Further, visual inspection of residual plots and Brusch-Pagan tests for these models (attention, valence, dominance, and believability) indicated heteroscedasticity, so additional models with robust standard errors, p values and CI are reported in Supplementary Table 6. The conclusions from the robust models do not differ to the bootstrapped models.

All models were run twice, once comparing both the physical health label and the mental health benefits label with the control group, and secondly excluding the control group to compare the mental health condition and the physical health condition with each other.

## Results

**Fig. 4:**
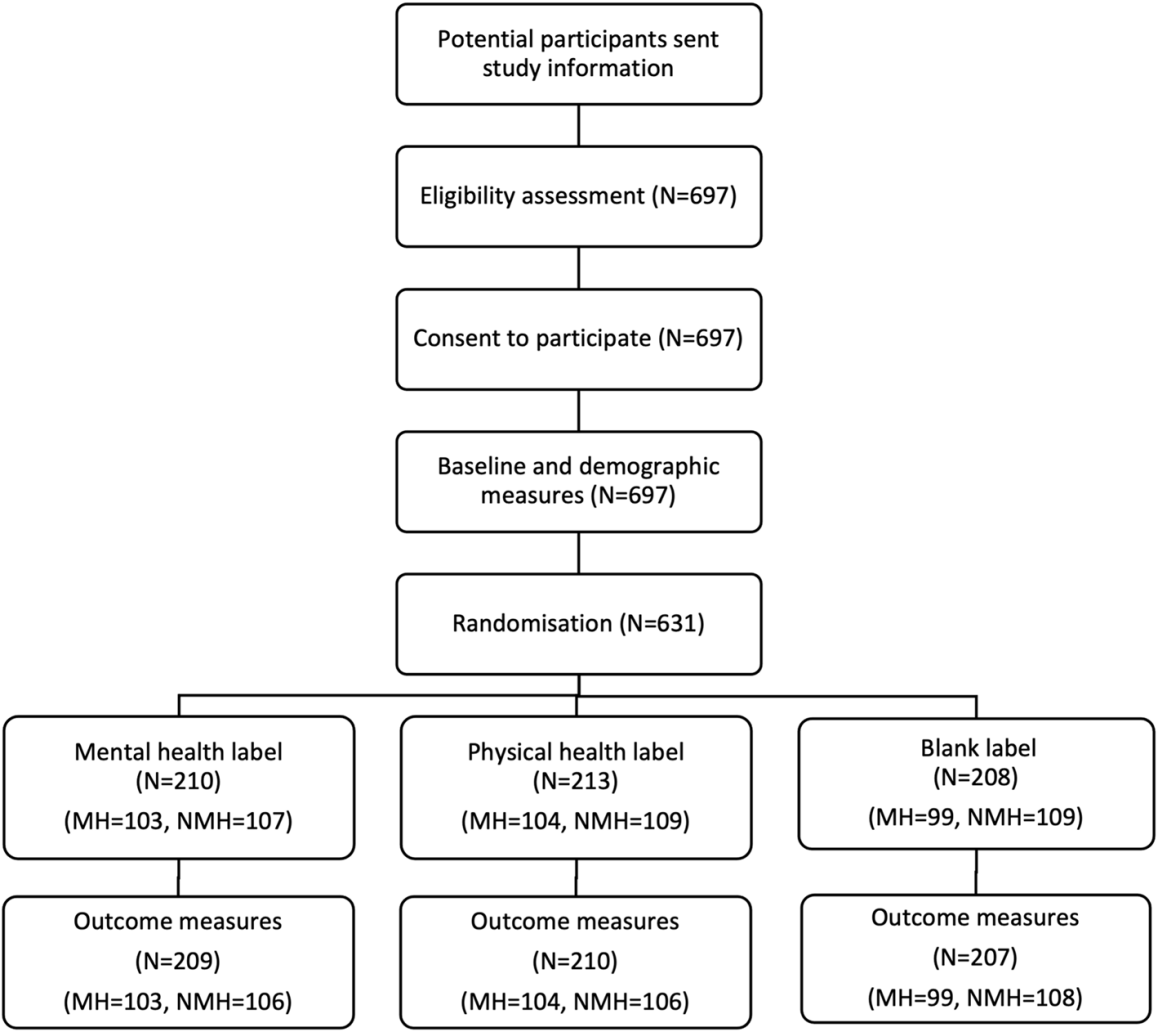
Study design and flow of participant through the study. *MH* = *mental health group, NMH* = *nonmental health group*

697 people consented to the study and completed baseline measures, which were reasonably balanced (Table 1). 66 people were not randomised either because they did not complete the survey or because we had reached the quota for a mental health status group. Thus, 631 were randomised, see Fig. 4 for study flow chart.

**Table 1:** Participant demographic and smoking characteristics at baseline, by experimental condition

	**Mental health**	**Physical health**	**Blank**
**N**	210	213	208
**Gender** N (%)			
Male	109 (51.9)	94 (44.13)	108 (51.9)
Female	98 (46.66)	115 (53.99)	99 (47.59)
Nonbinary/other/prefer not to say	3 (1.43)	4 (1.88)	1 (0.48)
**Age** M (SD)	37.91 (12.23)	40.34 (13.52)	39.07 (12.72)
**Ethnicity** N (%)			
White	185 (88.10)	185 (86.85)	188 (90.38)
Black	4 (1.90)	7 (3.29)	5 (2.40)
Asian	7 (3.33)	6 (2.82)	2 (0.96)
Mixed	5 (2.38)	5 (2.35)	1 (0.48)
Other	6 (2.86)	5 (2.35)	9 (4.33)
Prefer not to say	4 (1.90)	5 (2.35)	1 (0.48)
**Residence** N (%)			
UK	86 (40.95)	96 (45.07)	99 (47.60)
Outside UK	124 (59.05)	117 (54.93)	109 (52.40)
**Smoking information** M (SD)			
Baseline MTSS	3.3 (1.6)	3.2 (1.5)	3.1 (1.5)
FTND score	3.9 (2.4)	3.8 (2.4)	3.6 (2.4)
Previous quit attempts	2.1 (11.7)	2.5 (13.2)	2.5 (13.5)
**Mental health measure** M (SD)			
GAD7 score	7.0 (6.1)	7.2 (6.1)	6.9 (5.9)
PHQ9 score	8.6 (7.10)	8.1 (6.5)	7.9 (6.8)
SHAPS score	12.2 (2.4)	12.5 (2.0)	12.5 (2.3)

*MTSS* Motivation To Stop Scale*, FTND* Fagerstrom Test of Nicotine Dependence*, GAD7* Generalised Anxiety Disorder scale 7, *PHQ9* Patient Health Questionnaire 9, *SHAPS* Snaith-Hamilton Pleasure Scale

### Handling missing data

Five participants had missing outcome data for all outcomes, 626 participants had complete outcome data for the primary outcome and were analysed by original assigned groups (see Fig. 4). Some additional participants had missing data for secondary outcome measures (Supplementary Table 6). Participants with missing data were excluded from the analysis, as considering forced choice responses and the large sample size of the study, it was assumed that such minor missing data was unlikely to cause substantial bias in analysis.

### Primary research question

Motivation to stop smoking across the sample was low to moderate, the mean pre-MTSS score was 3.2 (SD = 1.5), and the mean post-MTSS was 3.3 (SD = 1.6). There was evidence for a difference in the effect of label condition on post MTSS scores between the mental health labels and blank labels, with the mental health label condition having higher post MTSS scores compared to the blank label condition (Table 2, model 1). There was evidence for a difference in the effect of label condition on post MTSS between the physical health label condition and blank label condition, with the physical health label condition having higher post MTSS scores compared to the blank label condition (Table 2, model 1). There was no evidence for a difference in the effect of label condition on post MTSS scores between the mental health label condition and physical health label condition (Table 2, model 2).

**Table 2:** Effect of experimental condition on post-viewing motivation to stop smoking scores^1^

Predictor	Coefficient	95% CI	*p*
Model 1: blank as reference^a^			
Pre-motivation to stop	0.96	0.93, 0.98	<.001**
Physical health label	0.11	0.01, 0.20	.031*
Mental health label	0.18	0.07, 0.30	.003*
Model 2: physical as reference^a^			
Pre-motivation to stop	0.96	0.93, 0.98	<.001**
Blank label	− 0.11	− 0.21, − 0.02	.02*
Mental health label	0.07	− 0.03, 0.18	.158

^*^ < 0.05, ** < 0.001

^a^bootstrapped 2000

*Does mental illness moderate the effect of label condition on motivation to stop?* There was no evidence that the effect of label condition on MTSS differed according to mental health status (*p* = 0.82 for interaction with mental health label condition, and *p* = 0.70 for interaction with physical health label condition, Supplementary Table 3). A sensitivity analyses controlling for baseline FTND scores did not change the results, (Supplementary Table 4).

*Does anhedonia moderate the effect of label condition on motivation to stop?* There was no evidence that anhedonia moderated the effect of label condition (*p* = 0.24 for interaction with mental health label and *p* = 0.72 for interaction with physical health label condition, Supplementary Table 5).

*Effect of label condition on the pre-specified Message Impact Framework variables.* Mental health labels increased intention to quit, post quitting self-efficacy scores, attention and decreased favourable beliefs about smoking, compared to the blank labels. There was no evidence for differences between the mental health labels and the physical health labels on intention to quit, quitting self-efficacy, attention, smoking beliefs, or arousal. Physical health labels were rated as less pleasant, more calming and less dominant than the mental health labels. However, scores for all conditions were around the mid-point of the SAM indicating a generally neutral response to the labels. Mental health labels were rated as less believable than the physical health labels. For more detail, the data are presented in Supplementary Tables 6, 7 and 8.

## Discussion

Motivation to stop smoking scores after viewing the intervention were higher in the mental health label group compared to the blank label group but there was no evidence that the effect differed between the mental health label and physical health label conditions. Furthermore, we did not find evidence that the effects of the labels on motivation to stop smoking was associated with symptoms of anxiety and depression or anhedonic traits. Mental health labels increased other elements in the Message Impact Framework but did so comparably to the physical health label condition.

The main findings of this study are consistent with evidence from Steinberg et al. [16] who found no difference in overall effect of positive mental health labels and physical health labels on motivation to quit smoking. Therefore, there is consistent evidence that gain-framed mental health labels may be as effective as physical health labels at increasing motivation to stop smoking.

Steinberg et al. [16] presented evidence in a longer format where messages consisted of 128 words across a page, which is not possible to use on tobacco packaging (16). The current study gives evidence that shorter concise labels suitable for use on tobacco packaging can also be effective at promoting motivation to stop smoking.

Steinberg et al. [16] found an interaction between messaging condition and mental health status; people with lifetime history of depression or anxiety reported greater motivation to quit in response to the mental health messages. However, there was no evidence of moderation of message effects by current symptoms in this study [16]. Our prior work found no differences in perceived effectiveness of loss-framed mental health and physical health messages between people with and without mental health problems. These studies could have been underpowered to detect moderate sized interaction effects, however taken together, there is no clear evidence that effects of mental health messages differ by mental health status or current symptoms; this should be further researched.

Many people who smoke believe that smoking improves mental wellbeing and this reflects their experience of smoking, whereas nearly all accept that smoking causes physical disease and quitting improves physical health [14, 32]. Prior research found negatively framed mental health warning labels are rated as less believable than physical health labels [14]. Similarly in this study, the positive mental health labels were rated as less believable than the physical health labels. This could be because the mental health labels assert new information that is opposite to current held beliefs. However, it is promising that the positive mental health labels increased key mechanisms for quitting behaviour compared to the blank labels, such as motivation, intention to quit and quitting self-efficacy, and had a similar size effect on motivation to stop smoking as the physical health labels. The mental health labels present novel anti-smoking messages, and this novelty could be more memorable or persuasive than the physical health labels, although potentially less believable.

Taken together increasing evidence on mental health warning labels suggest that despite commonly held beliefs about smoking, you could motivate cessation through simple messaging, and this suggests mental health labels may be useful in public health campaigns. Research investigating negatively framed mental health warnings on e-cigarettes found messages about stress, depression and anxiety produced similar ratings of intention to quit, and higher perceived effectiveness compared to current food and drug administration labels on addictiveness [23]. Our prior work found negatively framed mental health warnings for cigarette packaging were perceived as less effective than current physical health warnings, and had the potential to induce stigma and blame for people who smoke [38]. Therefore, labels depicting gain-framed mental health messages could be a more appropriate public health strategy than loss-framed mental health messages, although, future research should directly compare gain and loss framed mental health messages.

### Strengths and limitations

To our knowledge, this is the first study to investigate the effect of tobacco packaging depicting the benefits of quitting for mental health on motivation to stop smoking. The study used a controlled, experimental design enabling causal inferences to be made. A further strength of this study was that materials and outcome measures were informed by relevant PPI groups, indicating that gain-based messages would be less stigmatising in the context of mental health. However, a limitation of the current study is that the mental health messages are not compared to currently implemented loss-framed tobacco warning labels, therefore findings of this study have less real world relevance as we do not know if the gain-framed mental health labels are more effective than currently implemented tobacco warning labels. However, matching the experimental conditions on positive framing provided a more robust test of the topic of the warning, without concerns for using stigmatising mental health messages, as guided by PPI. Another limitation of this study is that we did not test the effects of the individual labels within each condition. The effects of label condition could have been driven by one of the four labels within each group, or a specific issue or feature of one label, but this was not tested. The results of this study warrant further research into the effectiveness of gain-framed mental health messages, including comparisons to current tobacco warning labels in circulation for greater real-world relevance.

The online design of the study does not represent tobacco packaging that people encounter when they purchase their cigarettes as participants are viewing packaging on a screen rather than buying actual tobacco products, thus it is uncertain to what extent this would generalise to tobacco packaging encountered in the real world. Future naturalistic studies could examine whether there is evidence that this short-term increase in motivation carries over into meaningful effects on population behaviour. The SHAPS measure of anhedonia reached a ceiling effect in this sample, indicating low levels of anhedonia, limiting the ability to detect moderating effects of anhedonia. Additionally, the study was powered to detect small effects for the primary analysis, so could have been underpowered to detect interaction effects for the secondary research questions. Another limitation of this study is the use of a self-report measures of smoking status and the absence of bio-verification, considering the potential for fraud in online studies it is difficult to be certain that all participants smoked tobacco weekly [39].

## Conclusions

Labels presenting evidence of the mental health benefits of quitting smoking increased motivation to stop smoking compared with blank packaging in a short-term online experiment. Gain-framed mental health labels could be an effective method to increase motivation to stop smoking, but further development and research is needed to estimate their effectiveness in real-world settings.

## Supplementary Information

Additional file 1.

Additional file 2.

## Data Availability

The datasets generated and/or analysed during the current study are available in the University of Bath repository 10.15125/BATH-01424. Scripts are available at 10.15125/BATH-01424, the protocol can be found at 10.17605/OSF.IO/3C75J.
