# Supplementary material for: What is the effect of presenting evidence of the mental vs physical health benefits of quitting smoking on motivation to stop smoking? An online randomised controlled experiment
Source: BMC Public Health. 2025 Jul 3;25:2331. doi: 10.1186/s12889-025-22795-0 (PMC12224690; doi:10.1186/s12889-025-22795-0)
Supplement: Supplementary file 2 — Additional file 2. [file 12889_2025_22795_MOESM2_ESM.docx]

**Supplementary Materials**

**What is the effect of presenting evidence of the mental vs physical health benefits of quitting smoking on motivation to stop smoking? An online randomised controlled experiment**

**Supplementary materials**

Table of Contents

[Table 1. Assessment schedule 3](#_Toc195104408)

[Table 2. Demographic characteristics by condition, split by mental health status. 4](#_Toc195104409)

[Table 3. Effect of experimental condition, mental health status and interaction effect on post-viewing motivation to stop smoking scores 5](#_Toc195104410)

[Table 4. Effect of experimental condition, FTND scores, mental health status, and interaction effect on post-viewing motivation to stop smoking scores 5](#_Toc195104411)

[Table 5. Effect of experimental condition, anhedonia and interaction effect on post-viewing motivation to stop smoking scores 6](#_Toc195104412)

[Table 6. Means and standard deviations for secondary outcomes by condition. 7](#_Toc195104413)

[Table 7. Secondary research question 3, associations between experimental conditions and each secondary outcome separately 8](#_Toc195104414)

[Table 8. Associations between experimental conditions and secondary outcomes; models with robust se, p values and CI due to heteroscedasticity. 9](#_Toc195104415)

# **Table 1. Assessment schedule**

|  |  | Enrolment | Baseline | Post |
| --- | --- | --- | --- | --- |
| Eligibility screen | Age | X |  |  |
|  | Smoking status | X |  |  |
|  | Language | X |  |  |
| Participant characteristics | Mental health status |  | X |  |
|  | Anhedonia |  | X |  |
|  | Country of residence |  | X |  |
|  | Gender |  | X |  |
|  | Education |  | X |  |
|  | Ethnicity |  | X |  |
|  | Nicotine dependence |  | X |  |
|  | Number of previous quit attempts |  | X |  |
| Primary outcome | Motivation to stop smoking |  | X | X |
| Secondary outcomes | Intention to quit smoking |  | X | X |
|  | Quitting self-efficacy |  | X | X |
|  | Smoking beliefs |  | X | X |
|  | Attention |  |  | X |
|  | Affective Reactions |  |  | X |
|  | Believability |  |  | X |

# **Table 2. *Demographic characteristics by condition, split by mental health status.***

|  | **Total** | **Mental NMH** | **Physical NMH** | **Blank  NMH** | **Mental  MH** | **Physical MH** | **Blank  MH** |
| --- | --- | --- | --- | --- | --- | --- | --- |
| N | 631 | 107 | 109 | 109 | 103 | 104 | 99 |
| **Gender** N(%) |  |  |  |  |  |  |  |
| Male | 311 (49.2) | 64 (59.8) | 54 (49.5) | 69 (63.3) | 45 (43.6) | 40 (38.4) | 39 (39.3) |
| Female | 312(49.4) | 43 (40.1) | 54 (49.5) | 40 (36.6) | 55 (53.3) | 61 (58.6) | 59 (59.5) |
| NonBinary/ Other/Prefer not to say | 8 (0.9) | 0 | 1 (0.9) | 0 | 3 (2.8) | 3 (2.8) | 1 (1.0) |
| **Age** M (SD) | 39.1 (12.9) | 40.2 (12.6) | 42.8 (14.1) | 42.8 (13.5) | 35.6 (11.5) | 37.9 (12.4) | 35 (10.4) |
| **Ethnicity** N (%) |  |  |  |  |  |  |  |
| White | 563 (89.2) | 96 (89.7) | 93 (85.3) | 96 (89.9) | 89 (86.4) | 92 (88.4) | 92 (92.9) |
| Black | 12 (1.9) | 2 (0.9) | 5 (4.5) | 3 (2.7) | 2 (1.9) | 2 1.9) | 2 (2.0) |
| Asian | 14 (2.2) | 4 (2.8) | 4 (3.6) | 2 (1.8) | 3 (2.9) | 2 (1.9) | 0 |
| Mixed | 12 (1.9) | 1 (1.9) | 2 (1.8) | 1 (0.9) | 4 (3.8) | 3 (2.8) | 0 |
| Other | 23 (3.6) | 3 (2.8) | 3 (2.7) | 4 (3.6) | 3 (2.9) | 2 (1.9) | 5 (5.0) |
| Prefer not to say | 10 (1.5) | 2 (1.8) | 2 (1.8) | 1 (0.9) | 2 (1.9) | 3 (2.8) | 0 |
| **Residence** N (%) |  |  |  |  |  |  |  |
| UK | 281 (44.5) | 42 (39.3) | 47 (43.1) | 52 (47.7) | 44 (42.7) | 49 (47.1) | 47 (47.4) |
| Outside UK | 350 (55.4) | 65 (60.7) | 62 (56.8) | 57 (52.2) | 59 (57.2) | 55 (52.8) | 52 (52.5) |
| **Smoking data** M (SD) |  |  |  |  |  |  |  |
| FTND score | 3.8 (2.4) | 3.6 (2.4) | 3.4 (2.4) | 3.3 (2.2) | 4.3 (2.4) | 4.1 (2.4) | 3.9 (2.5) |
| Previous quit attempts | 2.4 (12.8) | 0.6 (0.8) | 2.3 (12.8) | 1.3 (9.2) | 3.7 (16.6) | 2.6 (13.7) | 3.8 (16.9) |
| **Mental Health** M (SD) |  |  |  |  |  |  |  |
| GAD7 score | 7.0 (6.0) | 2.3 (2.3) | 2.7 (2.4) | 2.4 (2.1) | 12 (4.6) | 11.9 (5.0) | 11.8 (4.6) |
| PHQ9 score | 8.2 (6.8) | 3 (2.8) | 3.2 (2.7) | 2.9 (2.7) | 14.3 (5.5) | 13.3 (5.1) | 13.5 (5.5) |
| SHAPS score | 12.4 (2.2) | 13.1 (1.5) | 13.1 (1.2) | 13.1 (1.6) | 11.3 (2.7) | 11.8 (2.4) | 11.8 (2.7) |

*MTSS = Motivation stop stop scale, FTND = Fagerstrom test of nicotine dependence, GAD 7 =* Generalised Anxiety Disorder scale 7, PHQ9 = Patient Health Questionnaire 9, SHAPS = Snaith-Hamilton Pleasure Scale. MH = mental health group, NMH = nonmental health group

# **Table 3. Effect of experimental condition, mental health status and interaction effect on post-viewing motivation to stop smoking scores**

| **Predictor** | **Coefficient** | **95% CI** | ***p*** |
| --- | --- | --- | --- |
| Model 1: blank as reference*^1^* |  |  |  |
| Pre-motivation to stop | 0.96 | 0.93, 0.98 | <.001** |
| Physical health label | 0.13 | 0.01, 0.25 | .04* |
| Mental health label | 0.16 | 0.04, 0.31 | .009* |
| Mental health status | 0.02 | -0.12, 0.18 | .744 |
| Mental health status*physical health label | -0.04 | -0.24, 0.15 | .696 |
| Mental health status*mental health label | 0.03 | -0.21, 0.27 | .819 |
|  |  |  |  |
| Model 2: physical as reference*^1^* |  |  |  |
| Pre-motivation to stop | 0.96 | 0.93, 0.98 | <.001** |
| Blank label | -0.13 | -0.25, -0.01 | .041* |
| Mental health label | 0.04 | -0.11, 0.20 | .604 |
| Mental health status | -0.01 | -0.14, 0.11 | .832 |
| Mental health status*blank label | 0.04 | -0.16, 0.23 | .699 |
| Mental health status*mental health label | 0.06 | -0.14, 0.29 | .555 |
| *<0.05, ** <0.001, *^1^* bootstrapped 2000 |  |  |  |

# **Table 4. Effect of experimental condition, FTND scores, mental health status, and interaction effect on post-viewing motivation to stop smoking scores**

| **Predictor** | **Coefficient** | **95% CI** | **p** |
| --- | --- | --- | --- |
| Model 1 Sensitivity: blank as reference*^1^* |  |  |  |
| Pre-motivation to stop | 0.96 | 0.93, 0.98 | <.001** |
| Physical health label | 0.12 | 0.001, 0.25 | .047 |
| Mental health label | 0.16 | 0.04, 0.31 | .005* |
| Mental health status | 0.02 | -0.14, 0.16 | .784 |
| FTND score | 0.01 | -0.01, 0.03 | .614 |
| Mental health status*physical health label | -0.04 | -0.23, 0.16 | .691 |
| Mental health status*mental health label | 0.02 | -0.20, 0.27 | .793 |
|  |  |  |  |
| Model 2 Sensitivity: physical as reference*^1^* |  |  |  |
| Pre-motivation to stop | 0.96 | 0.93, 0.98 | <.001** |
| Blank label | -0.13 | -0.25, -0.01 | .041* |
| Mental health label | 0.04 | -0.11, 0.20 | .593 |
| Mental health status | -0.02 | -0.14, 0.10 | .745 |
| FTND score | 0.01 | -0.01, 0.02 | .608 |
| Mental health status*blank label | 0.04 | -0.15, 0.23 | .705 |
| Mental health status*mental health label | 0.07 | -0.15, 0.28 | .531 |
| *<0.05, ** <0.001, *^1^* bootstrapped 2000 |  |  |  |

# **Table 5. Effect of experimental condition, anhedonia and interaction effect on post-viewing motivation to stop smoking scores**

| **Predictor** | **Coefficient** | **95% CI** | ***p*** |
| --- | --- | --- | --- |
| Model 1: blank as reference*^1^* |  |  |  |
| Pre-motivation to stop | 0.96 | 0.93, 0.98 | <.001** |
| Physical health label | 0.11 | 0.01, 0.20 | .027* |
| Mental health label | 0.17 | 0.06, 0.29 | .002* |
| Anhedonia | 0.01 | -0.03, 0.05 | .641 |
| Anhedonia *physical health label | -0.01 | -0.06, 0.04 | .719 |
| Anhedonia*mental health label | -0.04 | -0.10, 0.02 | .244 |
| Model 2: physical as reference*^1^* |  |  |  |
| Pre-motivation to stop | 0.96 | 0.93, 0.98 | <.001** |
| Blank label | -0.11 | -0.21, -0.01 | .032* |
| Mental health label | 0.07 | -0.03, 0.18 | .182 |
| Anhedonia | 0.001 | -0.03, 0.03 | .890 |
| Anhedonia *blank label | 0.01 | -0.04, 0.05 | .712 |
| Anhedonia*mental health label | -0.03 | -0.08, 0.02 | .300 |
| *<0.05, ** <0.001, *^1^* bootstrapped 2000 |  |  |  |

# **Table 6. Means and standard deviations for secondary outcomes by condition.**

|  | **Pre** | | | | | **Post** | | | | |
| --- | --- | --- | --- | --- | --- | --- | --- | --- | --- | --- |
| **Outcome** | **N** | **Total** | **Mental health** | **Physical health** | **Blank** | **N** | **Total** | **Mental health** | **Physical health** | **Blank** |
| Intention to quit | 631 | 3.7 (2.5) | 3.9 (2.5) | 3.7 (2.4) | 3.5 (2.5) | 626 | 3.9(2.6) | 4.2 (2.7) | 4.0 (2.6) | 3.6 (2.5) |
| Quitting self-efficacy | 631 | 2.3 (0.8) | 2.4 (0.8) | 2.4 (0.8) | 2.3 (0.8) | 626 | 2.4(0.9) | 2.5 (0.9) | 2.4 (0.9) | 2.3 (0.9) |
| Smoking beliefs | 631 | 3.03 (0.6) | 3.02 (0.6) | 3.1 (0.6) | 3.02 (0.6) | 625 | 2.9(0.7) | 2.9 (0.7) | 3.0 (0.7) | 3.0 (0.7) |
| Attention | - | - | - | - | - | 625 | 3.5 (1.2) | 3.9 (1.0) | 4.0 (0.9) | 2.8 (1.2) |
| Valence | - | - | - | - | - | 621 | 4.8 (1.8) | 5.4 (1.9) | 4.7 (2.0) | 4.2 (1.4) |
| Arousal | - | - | - | - | - | 625 | 3.7 (1.8) | 3.9 (1.8) | 3.5 (1.7) | 3.7 (1.7) |
| Dominance | - | - | - | - | - | 625 | 5.2 (2.1) | 5.5 (2.0) | 5.0 (2.2) | 5.2 (1.9) |
| Believability | - | - | - | - | - | 625 | 4.0 (1.1) | 3.9 (1.1) | 4.5 (0.7) | 3.4 (1.1) |

# **Table 7. Secondary research question 3, associations between experimental conditions and each secondary outcome separately**

| **Outcome** | **Predictor** | **Coefficient** | **95% CI** | **p** |
| --- | --- | --- | --- | --- |
| Intention to quit^1^ | Model 1: blank as reference |  |  |  |
|  | Pre intention to quit | 1.00 | 0.97, 1.02 | <.001** |
|  | Physical health label | 0.29 | 0.13, 0.50 | <.001** |
|  | Mental health label | 0.25 | 0.10, 0.40 | .003* |
|  |  |  |  |  |
|  | Model 2: physical as reference |  |  |  |
|  | Pre intention to quit | 1.00 | 0.97, 1.02 | <.001** |
|  | Blank label | -0.29 | -0.48, -0.14 | .002* |
|  | Mental health label | -0.04 | -0.23, 0.13 | 0.622 |
|  |  |  |  |  |
| Quit self-efficacy^1^ | Model 1: blank as reference |  |  |  |
|  | Pre quitting self-efficacy | 0.96 | 0.93, 0.98 | <.001** |
|  | Physical health label | 0.03 | -0.03, 0.09 | .349 |
|  | Mental health label | 0.07 | 0.01, 0.14 | .040* |
|  |  |  |  |  |
|  | Model 2: physical as reference |  |  |  |
|  | Pre quitting self-efficacy | 0.96 | 0.93, 0.99 | <.001** |
|  | Blank label | -0.03 | -0.09, 0.03 | 0.366 |
|  | Mental health label | 0.04 | -0.03, 0.11 | 0.210 |
|  |  |  |  |  |
| Smoking beliefs^1^ | Model 1: blank as reference |  |  |  |
|  | Pre smoking beliefs | 0.94 | 0.89, 0.99 | <.001** |
|  | Physical health label | -0.06 | -0.13, 0.001 | .054 |
|  | Mental health label | -0.12 | -0.19, -0.06 | .002* |
|  |  |  |  |  |
|  | Model 2: physical as reference |  |  |  |
|  | Pre smoking beliefs | 0.94 | 0.89, 0.99 | <.001** |
|  | Blank label | 0.06 | -0.003, 0.12 | 0.058 |
|  | Mental health label | -0.06 | -0.13, 0.002 | 0.062 |
|  |  |  |  |  |
| Attention^1^ | Model 1: blank as reference |  |  |  |
|  | Physical health label | 1.16 | 0.96, 1.36 | <.001** |
|  | Mental health label | 1.10 | 0.86, 1.29 | <.001** |
|  |  |  |  |  |
|  | Model 2: physical as reference |  |  |  |
|  | Blank label | -1.17 | -1.37, -0.96 | <.001** |
|  | Mental health label | -0.09 | -0.29, 0.09 | 0.342 |
|  |  |  |  |  |
| Valence^1^ | Model 1: blank as reference |  |  |  |
|  | Physical health label | 0.56 | 0.24, 0.90 | <.001** |
|  | Mental health label | 1.20 | 0.89, 1.52 | <.001** |
|  |  |  |  |  |
|  | Model 2: physical as reference |  |  |  |
|  | Blank label | -0.56 | -0.90, -0.24 | <.001** |
|  | Mental health label | 0.64 | 0.28, 1.03 | <.001** |
|  |  |  |  |  |
| Arousal^1^ | Model 1: blank as reference |  |  |  |
|  | Physical health label | -0.20 | -0.54, 0.13 | .245 |
|  | Mental health label | 0.12 | -0.23, 0.46 | .479 |
|  |  |  |  |  |
|  | Model 2: physical as reference |  |  |  |
|  | Blank label | 0.20 | -0.12, 0.55 | .208 |
|  | Mental health label | 0.31 | -0.03, 0.66 | .069 |
|  |  |  |  |  |
| Dominance^1^ | Model 1: blank as reference |  |  |  |
|  | Physical health label | -0.13 | -0.53, 0.25 | .501 |
|  | Mental health label | 0.33 | -0.04, 0.70 | .075 |
|  |  |  |  |  |
|  | Model 2: physical as reference |  |  |  |
|  | Blank label | 0.14 | -0.25, 0.53 | .529 |
|  | Mental health label | 0.47 | 0.05, 0.88 | .028* |
|  |  |  |  |  |
| Believability^1^ | Model 1: blank as reference |  |  |  |
|  | Physical health label | 1.12 | 0.96, 1.30 | <.001** |
|  | Mental health label | 0.49 | 0.28, 0.70 | <.001** |
|  |  |  |  |  |
|  | Model 2: physical as reference |  |  |  |
|  | Blank label | -1.12 | -1.30, -0.95 | <.001** |
|  | Mental health label | -0.62 | -0.79, -0.46 | <.001** |
|  |  |  |  |  |
| *<0.05, ** <0.001, *^1^* bootstrapped 2000 | | | | |

# **Table 8. Associations between experimental conditions and secondary outcomes; models with robust se, p values and CI due to heteroscedasticity.**

| **Outcome** | **Predictor** | **Coefficient** | **95% CI** | **SE** | **p** |
| --- | --- | --- | --- | --- | --- |
| Attention^2^ | Model 1: blank as reference |  |  |  |  |
|  | Physical health label | 1.16 | 0.96, 1.34 | 0.10 | <.001** |
|  | Mental health label | 1.10 | 0.86, 1.30 | 0.11 | <.001** |
|  |  |  |  |  |  |
|  | Model 2: physical as reference |  |  |  |  |
|  | Blank label | -1.16 | -1.37, -0.96 | 0.10 | <.001** |
|  | Mental health label | -0.09 | -0.27, 0.10 | 0.10 | 0.371 |
|  |  |  |  |  |  |
| Valence^2^ | Model 1: blank as reference |  |  |  |  |
|  | Physical health label | 0.56 | 0.23, 0.89 | 0.17 | <.001** |
|  | Mental health label | 1.20 | 0.88, 1.52 | 0.16 | <.001** |
|  |  |  |  |  |  |
|  | Model 2: physical as reference |  |  |  |  |
|  | Blank label | -0.56 | -0.89, -0.23 | 0.17 | <.001** |
|  | Mental health label | 0.62 | 0.27, 1.02 | 0.19 | <.001** |
|  |  |  |  |  |  |
| Dominance^2^ | Model 1: blank as reference |  |  |  |  |
|  | Physical health label | -0.14 | -0.54, 0.27 | 0.20 | .507 |
|  | Mental health label | 0.33 | -0.04, 0.71 | 0.19 | .081 |
|  |  |  |  |  |  |
|  | Model 2: physical as reference |  |  |  |  |
|  | Blank label | 0.14 | -0.27, 0.54 | 0.20 | 0.507 |
|  | Mental health label | 0.47 | 0.06, 0.88 | 0.21 | 0.024* |
|  |  |  |  |  |  |
| Believability^2^ | Model 1: blank as reference |  |  |  |  |
|  | Physical health label | 1.12 | 0.94, 1.30 | 0.09 | <.001** |
|  | Mental health label | 0.49 | 0.29, 0.70 | 0.11 | <.001** |
|  |  |  |  |  |  |
|  | Model 2: physical as reference |  |  |  |  |
|  | Blank label | -1.12 | -1.30, -0.94 | 0.09 | <.001** |
|  | Mental health label | -0.63 | -0.80, -0.45 | 0.09 | <.001** |
|  |  |  |  |  |  |
| *<0.05, ** <0.001, ^2^ Robust SE reported due to heteroscedasticity | | | | | |
